# Construction of iron metabolism-related prognostic features of gastric cancer based on RNA sequencing and TCGA database

**DOI:** 10.1186/s12885-023-11569-9

**Published:** 2023-11-13

**Authors:** Xihong Liu, Junyu Ren, Ruize Zhou, Zhengqi Wen, Zhengwei Wen, Zihao Chen, Shanshan He, Hongbin Zhang

**Affiliations:** 1 Department of Oncology First Affiliated Hospital of Kunming Medical University, Kunming, Yunnan China; 2 Department of Pediatric Surgery First Affiliated Hospital of Kunming Medical University, Kunming, Yunnan 650032 P. R. China

**Keywords:** Gastric cancer, Prognostic signature, Iron metabolism-related, mRNA sequencing, TCGA Database

## Abstract

**Background:**

Researches have manifested that the disorder of iron metabolism is participated in Gastric cancer (GC), but whether iron metabolism-relevant genes (IMRGs) is related to the survival outcome of GC remain unknown.

**Methods:**

Eleven tumor as well as nine adjacent normal tissues from GC patients were underwent mRNA sequencing, and the The Cancer Genome Atlas Stomach Cancer (TCGA-STAD) datasets were acquired from the TCGA database. Cox analyses and least absolute shrinkage and selection operator (LASSO) regression were applied to build a IMRGs signature. The relationship between signature genes and the infiltration profiling of 24 immune cells were investigated using single-sample GSEA (ssGSEA). Meanwhile, the potential biological significance, genes that act synergistically with signature genes, and the upstream regulatory targets were predicted. Finally, the abundance of the signature genes were measured via the quantitative real-time PCR (qRT-PCR).

**Results:**

A IMRGs signature was constructed according to the expression and corresponding coefficient of *DOHH, P4HA3* and *MMP1* (The Schoenfeld individual test showed risk score was not significant with *P* values = 0.83). The prognostic outcome of patients in the high-risk group was terrible (*p* < 0.05). Receiver operating characteristic (ROC) curves confirmed that the IMRGs signature presented good efficiency for predicting GC prognosis (AUC > 0.6). The nomogram was performed well for clinical utilize (C-index = 0.60), and the *MMP1* expression significantly increased in the cohorts at age > 60 and Stage II-IV (*p* < 0.05). The positive correlation of *P4HA3* and *MMP1* expression as well as the negative correlation of *DOHH* expression with risk score (*p* < 0.0001) and worse prognosis (*p* < 0.05) were detected as well. Furthermore, 11 differential immune cells were associated with these signature genes (most *p* < 0.01). Finally, qRT-PCR revealed that the abundance of *DOHH, P4HA3* and *MMP1* were high in tumor cases, indicating the complex mechanism between the high expression of *DOHH* as a protective factor and the high expression of *P4HA3* and *MMP1* as the risk factors in the development of GC.

**Conclusion:**

An iron metabolism-related signature was constructed and has significant values for foretelling the OS of GC.

**Supplementary Information:**

The online version contains supplementary material available at 10.1186/s12885-023-11569-9.

## Introduction

Gastric cancer (GC) is a malignancy springing from the mucosal epithelial cells of stomach wall, which is one of the common digestive tract tumors. By 2020, the incidence of GC ranks fifth, and the mortality rate of GC ranks fourth [1]. At present, the treatment of gastric cancer is still mainly surgical treatment, combined with systemic chemotherapy, immunotherapy, targeted therapy, radiotherapy, and other auxiliary treatment mode. However, these treatments did not raise the five-year survival rate of patients with advanced GC [2]. In addition, GC patients are characterized by low early diagnosis rate, low radical resection rate and low 5-year survival rate [4–6]. Relevant research results show that advanced GC patients' median survival time is less than 1 year, and the five-year survival rate is about 18% [5–8]. The prognosis of metastatic GC patients is very poor, as the median of survival time is just 4–9 months [9]. Therefore, we need to find biomarkers with good prognosis prediction for GC, and to provide a new basis for clinical gene detection, targeted therapy and individualized treatment of GC.

Iron is an essential nutrient for promoting cell metabolism, proliferation and growth. The hydrogen peroxide was catalyzed by ferrous iron to generate reactive oxygen species through Fenton reavtion, which not only causes lipid and protein damage, but also oxidative damage DNA, inducing mutations and facilitating the emergence and preservation of tumors. Iron metabolism disorder is engaged in tumor occurrence, angiogenesis, invasion as well as metastasis, which is a general appearance in many tumors. Related studies have found that there are abnormal iron metabolism in lung cancer, prostate cancer, liver cancer, breast cancer, and, kidney cancer [10–13]. Some researches have also manifested that iron metabolism disorder is engaged in the process of gastric cancer, but whether it is linked to the prognosis of GC and the specific molecular mechanism are still unknown.

In this study, we first screened the DE-IMRGs from the GC expression profile of The Cancer Genome Atlas (TCGA) database, then constructed the prognostic signature of iron metabolism related GC, and probed the linkage between the immune cells and signature genes. Finally, the expression of signature genes was confirmed through external datasets and quantitative real-time PCR (qRT-PCR), which is a great significance for GC to explore potential therapeutic targets and molecular mechanisms.

## Materials and methods

### Data sourse

This research was allowed by the ethical committee of The First Affiliated Hospital of Kunming Medical University. All patients in this study signed written informed consent documents.

Specimen acquisition & sequencing: 11 tumor tissues and 9 normal tissues from GC patients were included in mRNA-seq analysis, where normal tissues were selected at least 1.0 cm from the tumor margin. These specimens were all gathered from The First Affiliated Hospital of Kunming Medical University and were freshly frozen and reserved at -80 °C immediately after surgery. Afterward, the total RNA from tissue cases was extracted using TRIzol reagent (Invitrogen, CA, USA). The RNA integrity was assessed by Bioanalyzer 2100 (Agilent, CA, USA) and then a final cDNA library with the average insert size of 300 + 50 bp by PCR was estimated. Finally, the mRNA sequencing was run relying on the illumina Novaseq™ 6000 (LC Bio Technology CO.,Ltd. Hangzhou, CHN).

Collection of TCGA data and iron metabolism-relevant genes (IMRGs): Additionally, the mRNA data, clinicopathological data and DNA methylation information from the TCGA Stomach Cancer (STAD) datasets were regained from the TCGA database (https://www.cancer.gov/ccg/research/genome-sequencing/tcga, accessed on 13 October 2017), including 373 tumor cases and 32 normal cases. The 428 IMRGs cohorts (v7.4) was downloaded from the Molecular Signatures Database (MSigDB v2023.1.Hs) database (https://www.gsea-msigdb.org/gsea/msigdb) with the keywords of Iron metabolism and we retrieved 428 IMRGs.

### Identification of differentially expressed genes (DEGs)

The mRNA data from the sequencing data were selected for identification of the DEGs through edgeR package [14, 15]. Similarly, the mRNA data from the 373 tumor and 32 normal cases of TCGA database were used to identify the DEGs via edgeR package [14, 15]. The DEGs were displayed in the volcano map by ggplot2 package. The overlapping genes of the IMRGs, the DEGs of TCGA and the DEGs of sequencing data were selected by Venn tool. These overlapping genes were DE-IMRGs.

### The correlation between DEGs and CpG site methylation

The CpG site methylation levels of DE-IMRGs were extracted from the TCGA, and the relationship were estimated between the DE-IMRGs and CpG site methylation levels via psych [16] in R (|cor|> 0.25).

### Construction and evaluation of a IMRGs signature

The univariate and multivariate cox regression analyses were firstly run to select survival-related genes using survminer R package (version 0.4.8). The prognostic genes were further confirmed using LASSO regression algorithm via glmnet R package (version 4.1–3). After that, the 345 cancer cases with complete survival data in the TCGA database were classified into two cohorts, namely training cohort (242) and validation cohort (103), according to 7:3. The formula for calculation of risk score was ‘h0(t)*exp(β1X1 + β2X2 + … + βnXn)’ via survival in R. The Global Schoenfeld residual test was employed for checking Proportional Hazard (PH) assumptions and estimating the partial residuals estimated by the Cox proportional hazards model via a residual plot. Further, the cases were separated into low- and high-risk group based on the median of risk-score. Besides, the Kaplan–Meier survival curve as well as the ROC curve were produced to evaluate the signature, and were drawn via survival and the survivalROC in R software.

### Independent prognostic analysis of IMRGs signature

All of the clinicopathological factors of 345 cancers with complete clinical information were subjected to the model for COX independent prognostic analysis to assess the independent clinical prognostic factors. Afterward, the nomogram was built for GC patients to predict the survival of via the R package rms.

### Correlation analysis between the IMRGs signature and clinical characteristics

The clinical information (Age, gender, pathological M, N, T stage, tumor stage and treatment type), survival information of GC data in the TCGA database were collected, as well as the classification of low- and high-risk groups and signature genes. Heatmap of signature genes expression between different clinical subgroups was displayed using pheatmap (version 1.0.12) and magrittr (version 2.0.1) and the statistic results were exhibited with the box plots using Wilcoxon test. And meanwhile, the survival differences of 345 patients with GC in high- and low- expression patterns of signature genes was analyzed using Kaplan–Meier survival analysis.

### Immune infiltration analysis

The ssGSEA algorithm and Wilcoxon test were applied to estimate the infiltration profile of immune cell and the differences in immune infiltration between two risk subgroups. Meanwhile, the relation between the 24 immune cells and signature genes were investigated based on the ssGSEA algorithm.

### Gene Set Enrichment Analysis (GSEA)

GSEA were conducted for the potential biological significance and classical functions involving signature genes in GC. Using ‘c5.go.v7.4.entrez.gmt’ (GO) and ‘c2.cp.kegg.v7.4.entrez.gmt’ (KEGG) downloaded from the GSEA website (http://www.gsea-msigdb.org/gsea/msigdb, accessed on 6 September 2023) as the background gene set, the high- and low- expression groups of each signature gene were divided based on the median value of the expression values for GSEA using clusterProfiler [17] (version 4.0.2) and org.Hs.eg.db (version 3.13.0), and threshold was set to | NES |> 1, NOMP < 0.05, *q* < 0.25.

### Collaborative gene analysis of signature

In order to study the genes that cooperate with the signature genes, R package psych [16] was run to count the pearson correlation between the three signature genes with all genes, and then performed the correlation analysis according to |Pearson coefficient value|> 0.6 with FDR < 0.05 threshold.

The function annotation of different collaborative genes were analyzed via the ClusterProfiler [17–20]. Additionally, enrichment analysis was performed separately for each collaborative gene based on a significance threshold *p* < 0.05 and visualized by ggplot2 in R.

### Transcription factors (TFs)-miRNA-mRNA network of signature genes

In order to reveal which miRNAs targeting signature genes and which TF interacting with signature genes may be involved in the prognosis in GC, TRRUST database (http://www.grnpedia.org/trrust, accessed on 6 September 2023) and miRWalk database (http://mirwalk.umm.uni-heidelberg.de/, accessed on 6 September 2023) were utilized for prediction analysis. The TF-miRNA-mRNA network was constructed and visualized using Cytoscape software.

### qRT-PCR

The total RNA of the tumor cases (10) and the normal cases (10) were extracted using TRIzol Reagent (Invitrogen, CA, USA). The sweScript RT I First strand cDNA SynthesisAll-in-OneTM First-Strand cDNA Synthesis Kit (Servicebio, WuHan, CHN) was used for reverse transcription. The primer sequences were showed in the Table S1. The reference gene GAPDH was used in qRT-PCR experiments. Finally, the relative abundance of signature genes was detected by the 2xUniversal Blue SYBR Green qPCR Master Mix (Servicebio, WuHan, CHN) and standardized with the 2^−ΔΔct^ method.

## Results

### Identification of DEGs

**Fig. 1:**
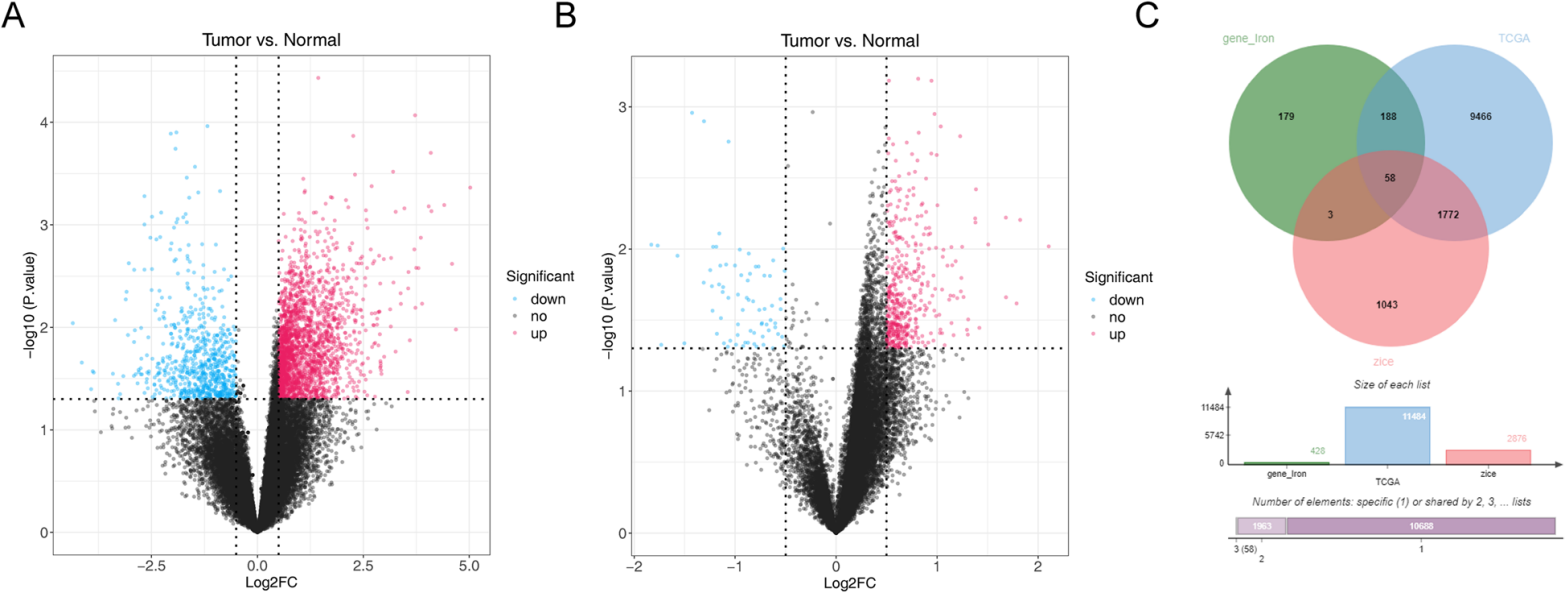
Identification of DE-IMRGs. **A** The volcano plot shows the DEGs between tumor and normal in the sequencing data. **B** volcano plot shows the DEGs between tumor and normal in TCGA database. **C** The Venn plot shows the DEGs from the sequencing data and TCGA database and IMRGs

In the sequencing data, 11,481 DEGs were screened from the tumor and normal cases (Fig. 1A). In TCGA database, 5062 DEGs were screened from the tumor and normal cases (Fig. 1B). Finally, 58 DE-IMRGs were obtained from 5062 DEGs of TCGA, 11,481 DEGs of sequencing data and 428 IMRGs (Fig. 1C).

### The linkage between DEGs and methylation of CpG site

Ten DEGs whose expression were associated with CpG sites were obtained to explore the potential regulation of DNA methylation on gene expression (Table 1), including *SLC11A1*, *FANCG*, *CCND1*, *ALKBH2*, *CDK5RAP1*, *CDO1*, *HMBS*, *NTHL1*, *FXN* and *KIF23*.

**Table 1:** DEG-CpGs

Symbol	logFC-exp	cor	*p*-value
SLC11A1	1.603420664	0.5011	0.036827
FANCG	0.669374024	-0.3843059	2.87E-13
CCND1	0.578168838	-0.3466985	6.35E-11
ALKBH2	0.705188653	-0.3018947	1.65E-08
CDK5RAP1	0.588938787	-0.2978591	2.6E-08
CDO1	-1.39378896	-0.2903042	6E-08
HMBS	0.889997626	-0.2693556	5.39E-07
NTHL1	0.755057612	-0.2686109	5.81E-07
FXN	0.698112433	-0.2547962	2.23E-06
KIF23	1.595989666	-0.2520228	2.9E-06
PLOD3	0.856492524	-0.2372911	1.1E-05
GLRX3	0.51508111	-0.2336994	1.51E-05
RAD51	1.522265757	-0.2295502	2.15E-05
ATP6V1C1	0.516632482	-0.2265069	2.78E-05
PUS1	0.939137431	-0.2249831	3.15E-05
FANCI	1.382012951	-0.2147057	7.27E-05
XRCC2	1.635300181	-0.2004757	0.000217
P4HA3	1.973571416	0.1896872	0.000473
ATP6V1C2	1.244956964	-0.183886	0.000707
CYP27B1	1.336683143	-0.183343	0.000733
UBE2T	1.532447267	-0.1635036	0.002646
PRIM2	0.761406132	-0.1614566	0.002997
PALB2	0.654905467	-0.1583963	0.003602
HYAL2	0.650940956	-0.1528398	0.00499
POLA1	0.703782535	-0.1504755	0.005714
ALOX12B	1.390150295	0.1495621	0.006019
CYP4B1	-1.92512197	-0.1439937	0.008208
SCARA5	-1.92784436	0.1277368	0.01916
CCNB1	1.735808613	-0.1267312	0.02014
REP15	-2.2991481	-0.1202464	0.02753
RTEL1	0.967633929	-0.1037375	0.05749
ABCE1	0.893800372	0.1032949	0.05857
POLD1	0.879078201	-0.0991999	0.06936
TMPRSS6	-1.56762532	0.09883517	0.0704
RFWD3	0.760886638	-0.09304779	0.08858
FANCB	1.564025495	-0.09022061	0.09874
FANCA	1.248125352	-0.08374725	0.1255
PLOD1	0.844171575	-0.07410696	0.1754
COL7A1	1.812713075	-0.06719322	0.2193
DNA2	0.987347716	0.06629113	0.2255
FANCM	0.640403824	0.06592408	0.2281
PPEF1	1.314169478	0.06155626	0.2605
DOHH	0.680727916	-0.05825401	0.287
MMP1	1.558222571	-0.05223964	0.3398
CYP26B1	1.312969294	-0.04155381	0.4477
PPAT	1.01160099	0.03998513	0.4651
BRIP1	0.876330825	-0.03070913	0.5748
FANCE	0.816669612	-0.02599784	0.6349
YARS2	0.514504526	-0.00627551	0.9088
BRCA1	1.050006084	0.000138788	0.998

### Construction and evaluation of a IMRGs signature

**Fig. 2:**
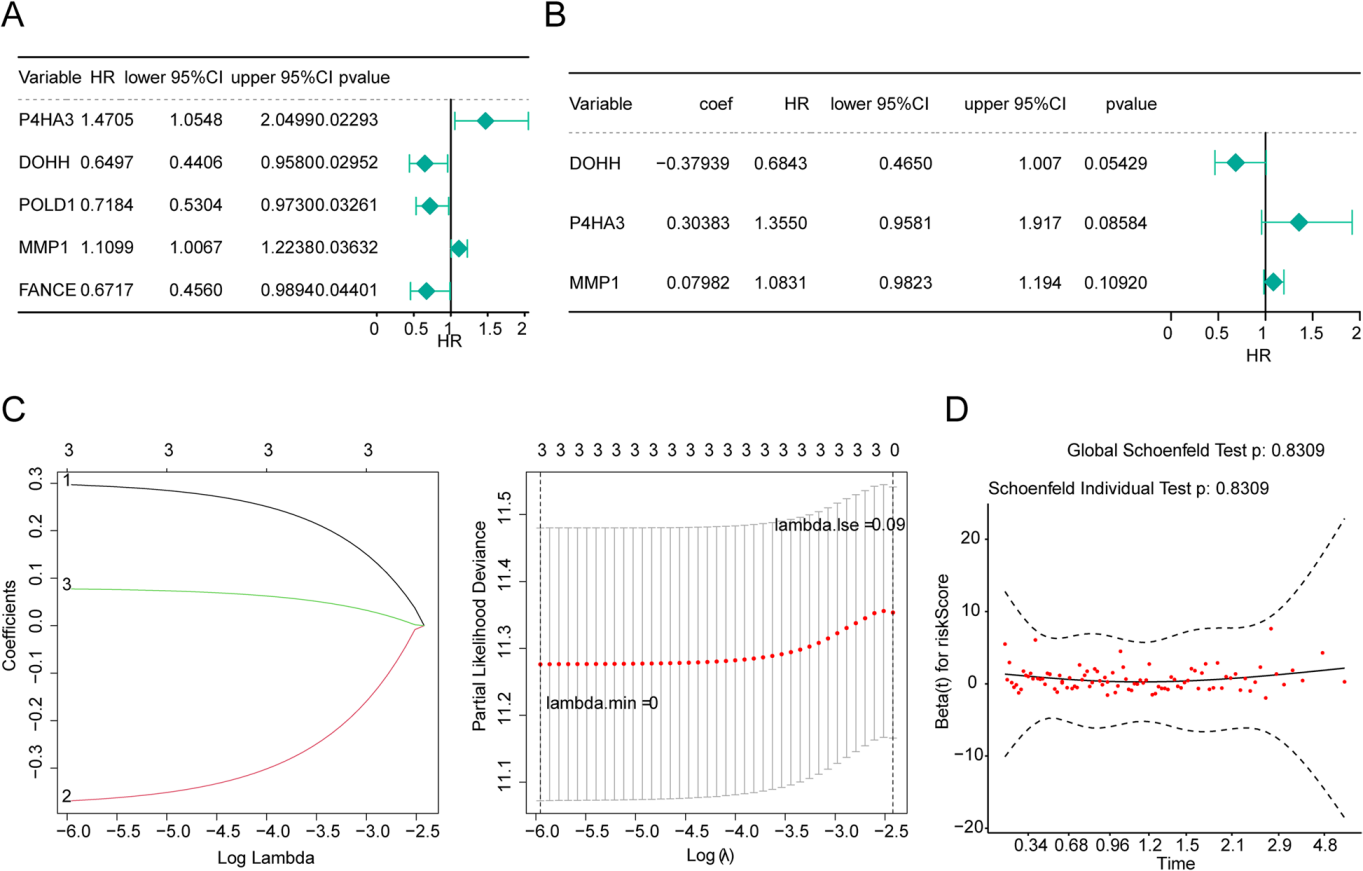
Construction and evaluation of a IMRGs signature. **A** Univariate Cox regression analysis selected 5 prognosis related genes (*P4HA3*, *DOHH*, *POLD1*, *MMP1* and *FANCE*); **B** Multivariate Cox regression analysis selected 3 prognosis related genes (*DOHH*, *P4HA3* and *MMP1*) as signature genes. **C** LASSO coefficients profiles (left) to determine the number of factors and cross-validation diagram (right) for tuning parameter selection in the least absolute shrinkage and selection operator (LASSO) model. From left to right along the x-axis, with the increases of lambda, the compression parameter increases and the absolute value of the coefficient decreases. The number on top are the number of variables with non-zero regression coefficients in the LASSO model. Variables with non-zero coefficients are important features for our screening. **D** The Schoenfeld individual test showed *P* values = 0.83

The 58 DE-IMRGs of the TCGA database were imported to univariate COX regression and five prognosis related genes (*P4HA3*, *DOHH*, *POLD1*, *MMP1* and *FANCE*) were obtained (Fig. 2A). These five genes were further imputed in multivariate Cox regression analysis, indicating *DOHH* (coef = -0.38, HR = 0.68, *p* = 0.05), *P4HA3* (coef = 0.30, HR = 1.36, *p* = 0.09) and *MMP1* (coef = 0.08, HR = 1.08, *p* = 0.11) were selected as signature genes (Fig. 2B). Further, the LASSO algorithm confirmed the importance of three prognostic genes, and *DOHH*, *P4HA3*, and *MMP1* were involved in the Cox proportional hazards model for construction of the IMRGs signature (Fig. 2C). The Schoenfeld individual test showed risk score was not significant with *P* values = 0.83 (Fig. 2D), suggesting the PH assumption of the IMRGs signature remained inviolate.

**Fig. 3:**
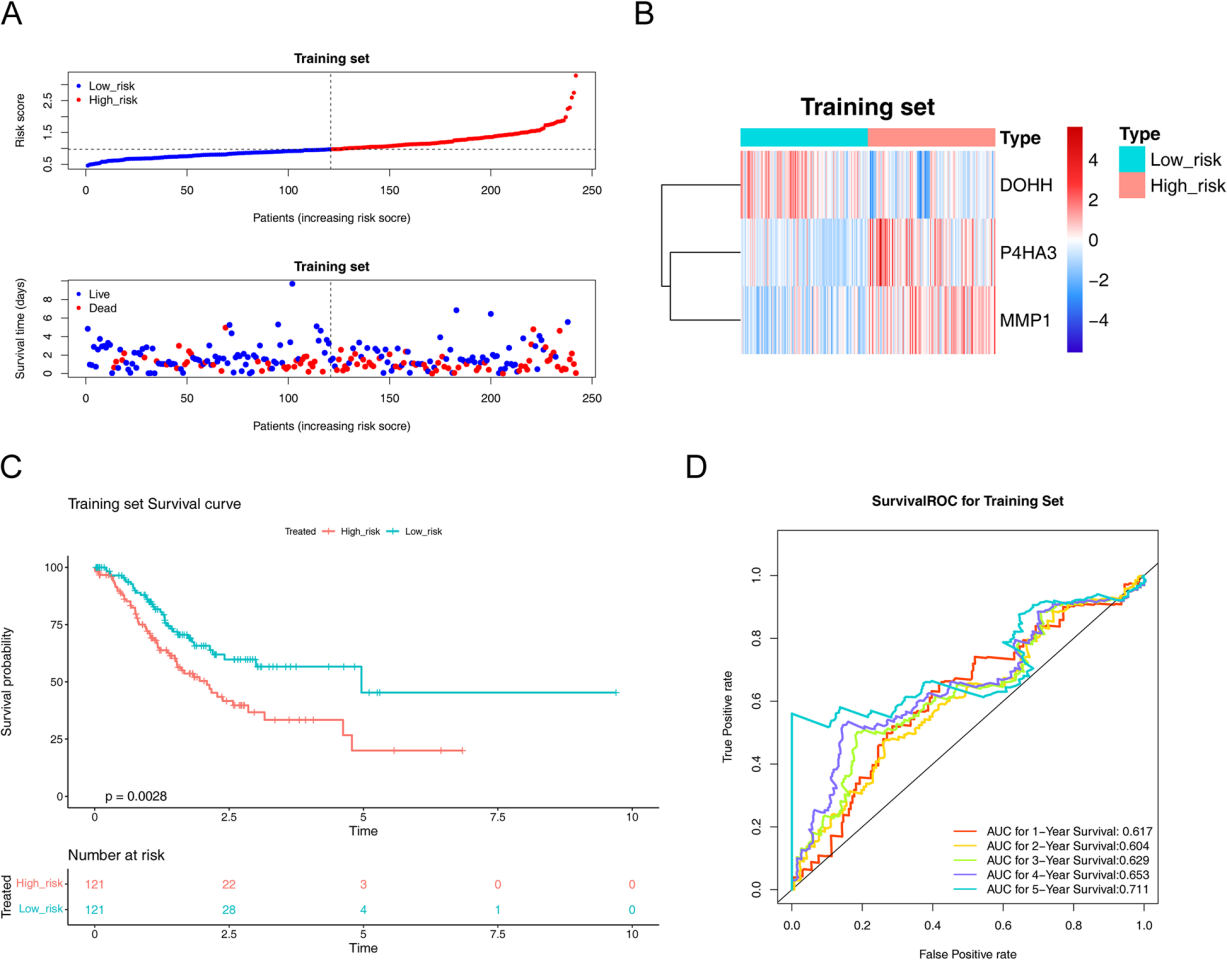
Construction of the signature genes prognostic signature based on signature genes in the training cohort. **A** The distribution of the risk score, OS. **B** Heatmap of the signature genes correlated with risk-score. **C** Kaplan Meier analysis of two risk groups. **D** AUC value of prediction performance of the model for survival rate (1, 2, 3, 4 and 5 year)

The patients in training cohort were classified into low- and high-risk groups (Fig. 3A). In addition, the signature genes of *P4HA3* and *MMP1* were positively linked to risk-score, but the *DOHH* was negatively associated with risk-score (Fig. 3B). The Kaplan–Meier curve showed that patients with higher risk had a poorer prognosis with *p* < 0.01 (Fig. 3C). In details, the AUCs for OS (1-, 2-, 3-, 4- and 5-years) were 0.617, 0.604, 0.629, 0.653 and 0.711 (Fig. 3D).

**Fig. 4:**
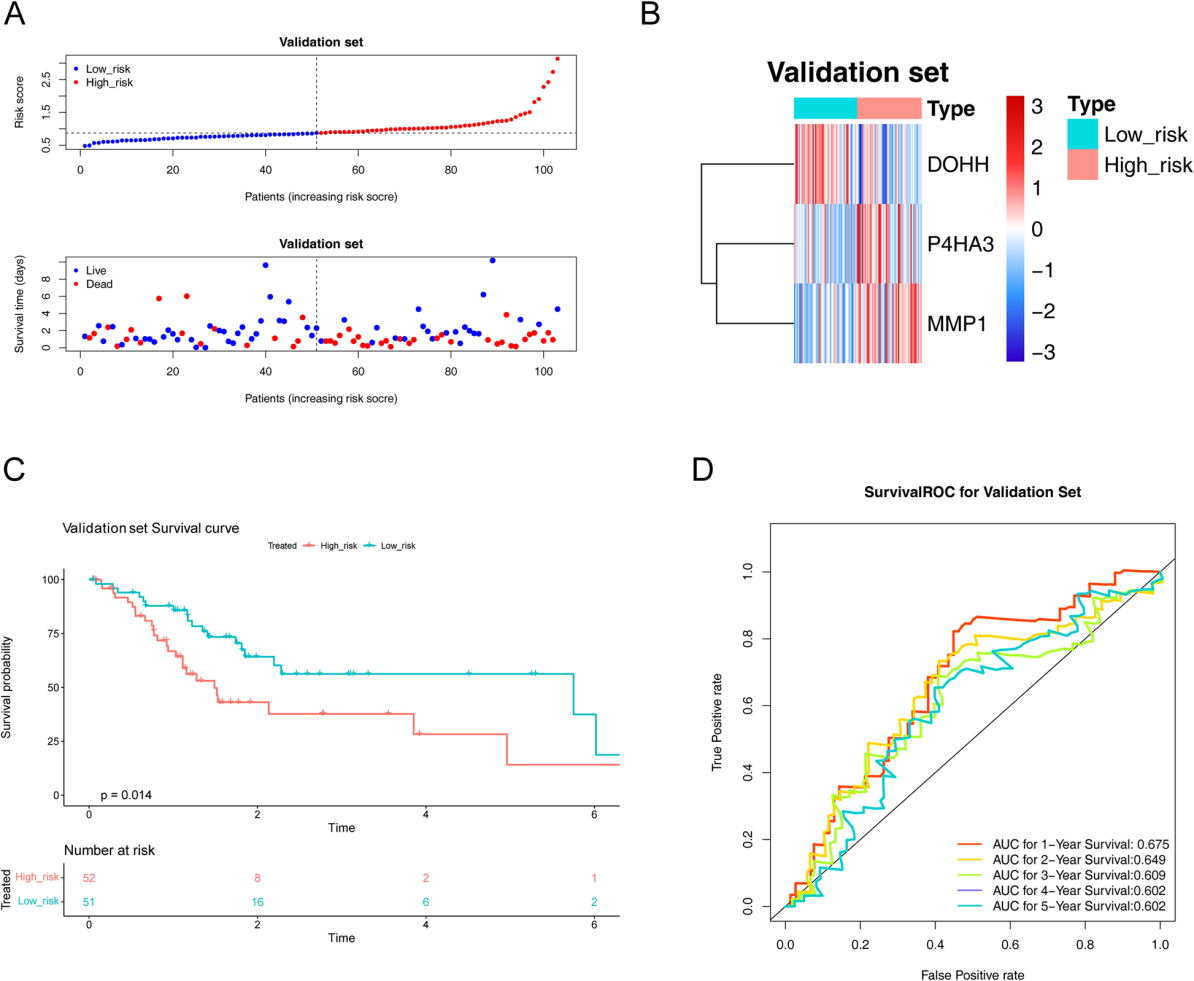
Construction of the signature genes prognostic signature in the validation cohort. **A** The distribution of the risk score, OS. **B** Heatmap of the signature genes correlated with risk-score. **C** Kaplan Meier analysis of two risk groups. **D** AUC value of prediction performance of the model for survival rate (1, 2, 3, 4 and 5 year)

Likewise, the GC patients of validation cohort were segmented into low- and high-risk groups (Fig. 4A). The correlation of risk-score and signature genes (Fig. 4B), and the result of Kaplan–Meier curve were similar with training cohort (*p* = 0.01) (Fig. 4C). Moreover, the AUCs for 1-year was 0.675; 2-years was 0.649; 3-years was 0.609; 4-years was 0.602 and 5-years was 0.602 (Fig. 4D).

### Independent prognostic analysis of IMRGs signature

**Fig. 5:**
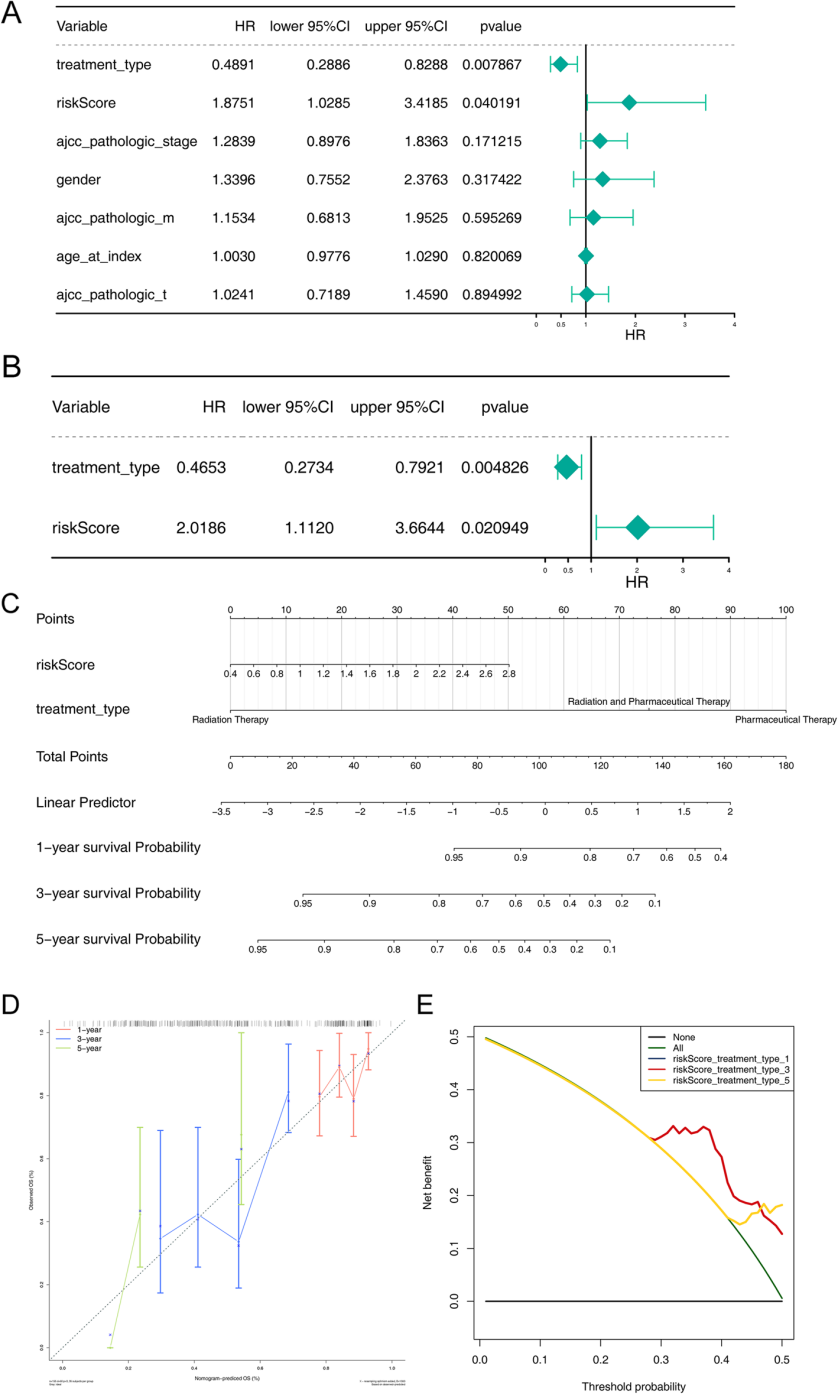
Building and evaluation of a nomogram model linked to signature. **A** Univariate Cox regression analysis; **B **Multivariate Cox regression analysis to select independent prognostic factors. **C** The nomogram model comprised of independent prognostic factors; **D** Calibration curves of OS (1-, 3-, and 5-year) predicted by the nomogram; **E** The DCA curves to show the expected net benefits based on the nomogram prediction at different threshold probabilities. None: assume an event will occur in no patients (horizontal black line); All: assume an event will occur in all patients (green line)

The 345 patients of TCGA with complete information including treatment type, risk-score, ajcc pathologic stage, gender, ajcc pathologic m, age at index and ajcc pathologic t were included for univariate Cox regression analysis. As shown in Fig. 5A, treatment type and risk-score were related with risk model. Moreover, treatment type and risk-score were selected as independent prognostic factors by multivariate Cox regression analysis (Fig. 5B). Next, treatment type and risk-score were included to establish a nomogram and the C-index of nomogram model was 0.60 (Fig. 5C). Furthermore, the calibration effect of 1- and 3-years in calibration curve were performed well (Fig. 5D). Decision curve analysis (DCA) shows that the nomogram model achieves better net benefit than 1-year OS rate (Fig. 5E).

### Correlation analysis between the IMRGs signature and clinical characteristics

**Fig. 6:**
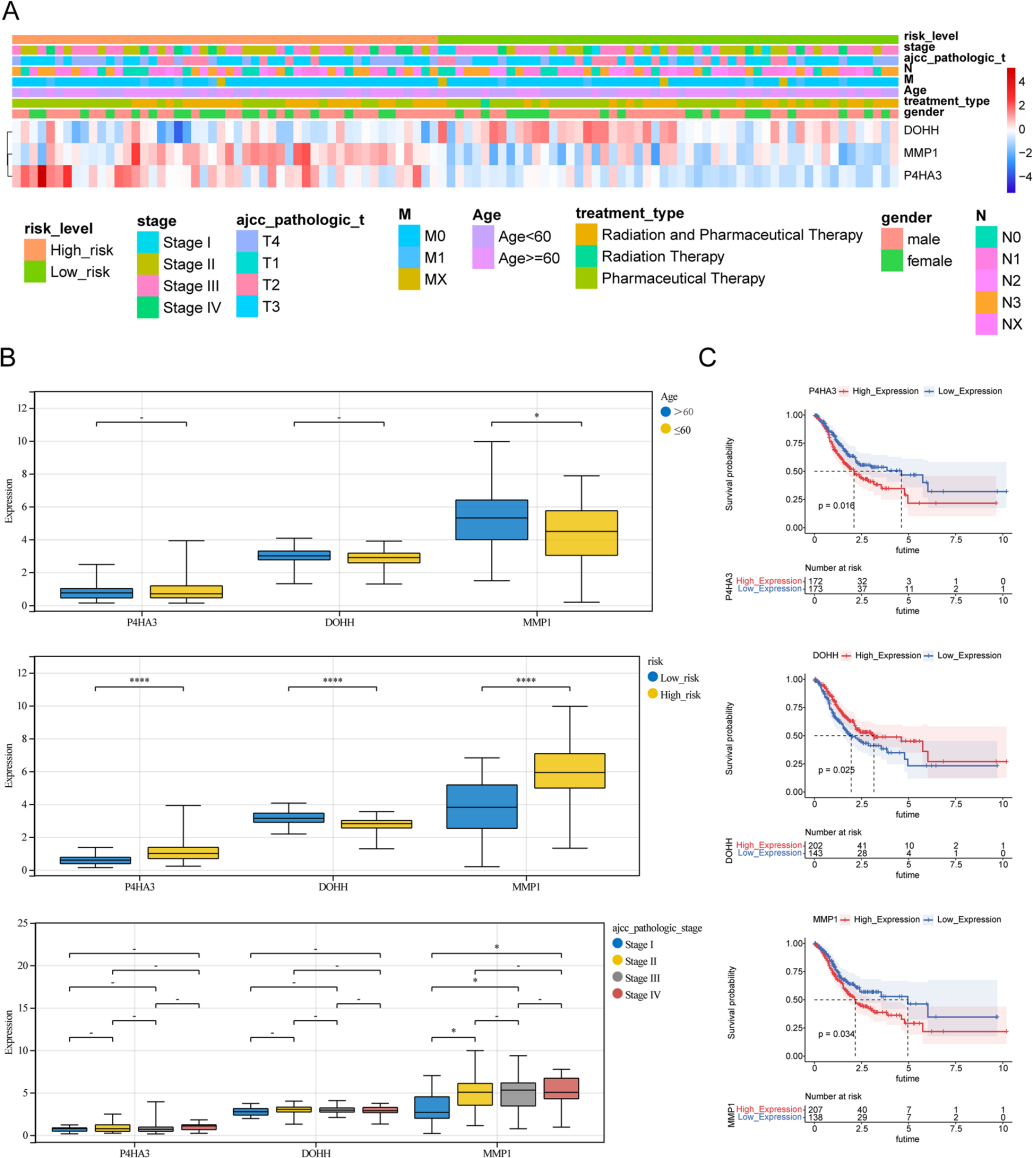
The linkage between the IMRGs signature and clinical characteristics. **A** Heatmap for the expression of three signature genes in different clinical sub-groups. **B** The boxplot for the expression of three signature genes in different clinical sub-groups (age, risk score, tumor pathologic stage); **C** Kaplan Meier analysis of high- and low- expression groups of each signature gene

The heatmap and box plots showed that the expression of the *P4HA3* and *MMP1* were high in GC patients of high-risk group. However, the abundance of the *DOHH* was high in GC of low-risk group (*p* < 0.0001) (Fig. 6A-B). And meanwhile, patients with high expression of *P4HA3* and *MMP1* had worse prognosis than that with low expression, otherwise, individuals with high expression of *DOHH* had a high survival probability (*p* < 0.05) (Fig. 6C), consist with the results generated above. Further, it can be seen that the expression of *MMP1* was related to the age and tumor stage of GC patients, that is, the *MMP1* expression significantly increased in the cohorts at age > 60 and Stage II-IV (*p* < 0.05) (Fig. 6B).

### Immune infiltration analysis

**Fig. 7:**
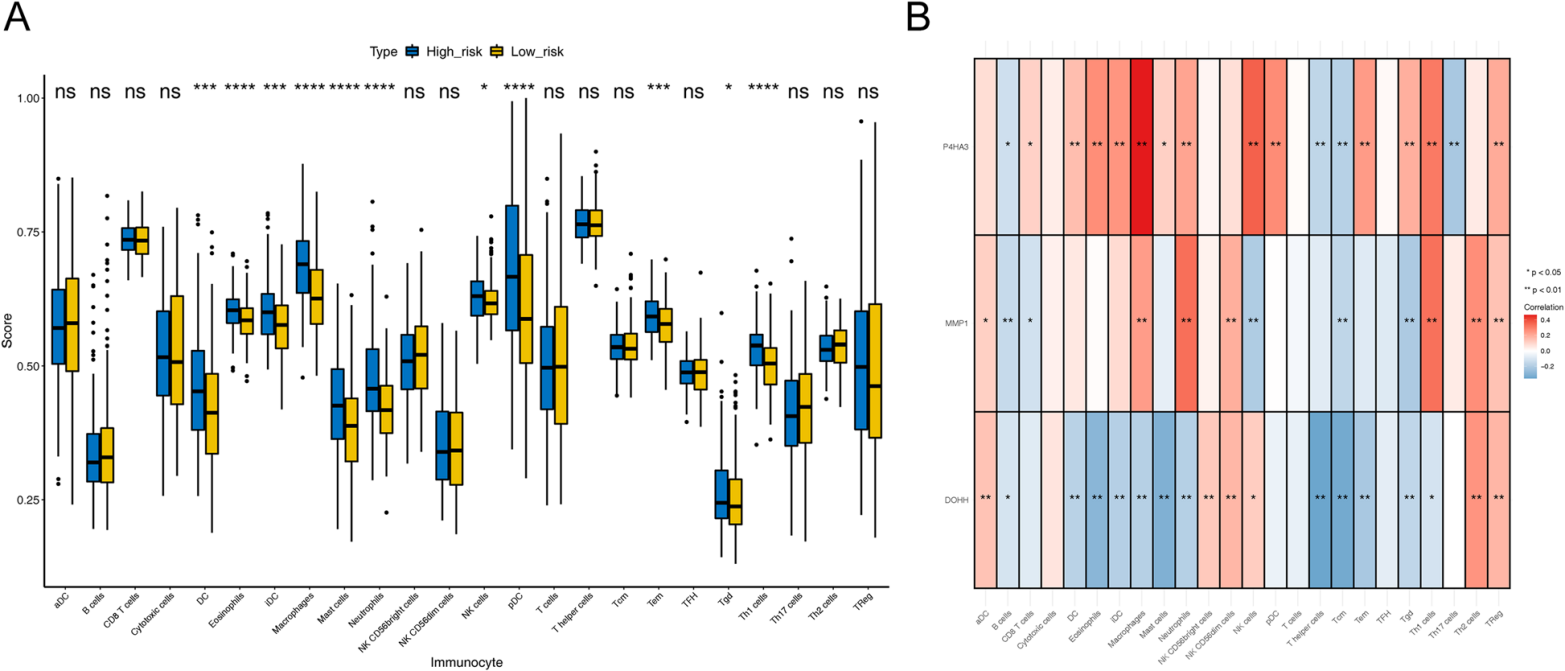
The imparity of immune infiltration in patients with differing risks. **A** Comparison of 24 immune cells between high-risk group and low-risk group; **B** The linkage heat map between the signature genes and 24 immune cells

For studying the imparity of immune infiltration in patients with differing risks, the spearman correlations for 24 immune cells were calculated. The 11 immune cells were different between the two risk GC subgroups (Fig. 7A). The relationship between the signature genes and 24 immune cells and signature genes shown that *P4HA3* was significantly associated with 17 immune cells; *MMP1* was significantly associated with 12 immune cells; *DOHH* was significantly related to 18 immune cells (Fig. 7B).

### GSEA of signature genes

**Fig. 8:**
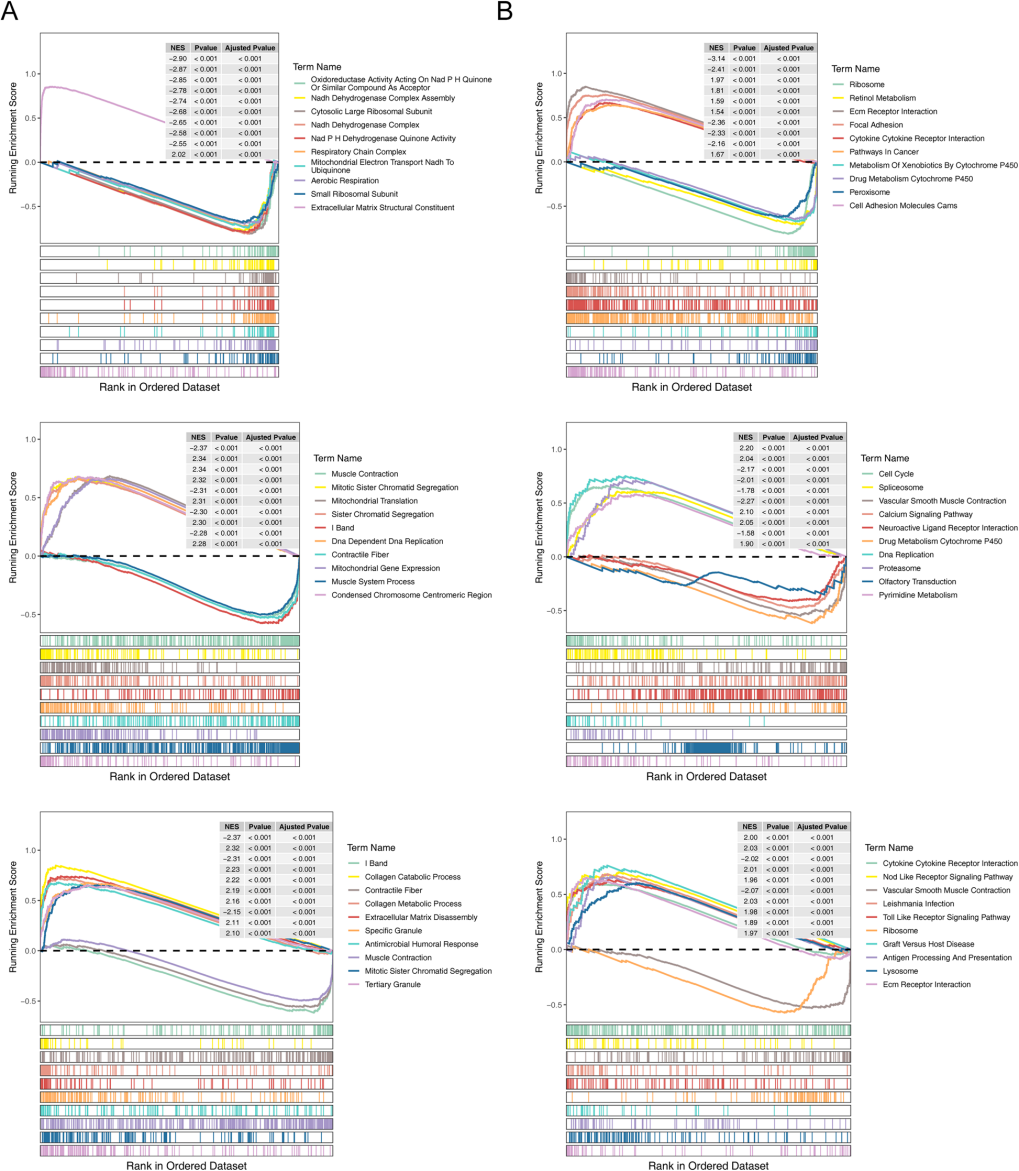
Single gene set enrichment analysis (GSEA) for three signature genes, including *P4HA3, DOHH, MMP1*. **A** GO analysis; **B** KEGG analysis

For the biological functions and signaling pathways involving signature genes, GSEA were employed under the GO and KEGG analysis (Fig. 8A-B). It was indicated that *P4HA3* was mainly enriched in the cytokine-cytokine receptor, interaction pathways in cancer and the functions of extracellular matrix structural constituent. *DOHH* is mainly functionally related to the KEGG pathways of cell cycle, calcium signaling pathway, as well as the GO functions of DNA replication and muscle contraction. *MMP1* might be relevant to the enrichment of Toll-like/Nod-like receptor signaling pathways and various catabolic processes. Besides, it was observed that ascular smooth muscle contraction term was common to *DOHH* and *MMP1.*

### Collaborative gene analysis of signature genes

**Fig. 9:**
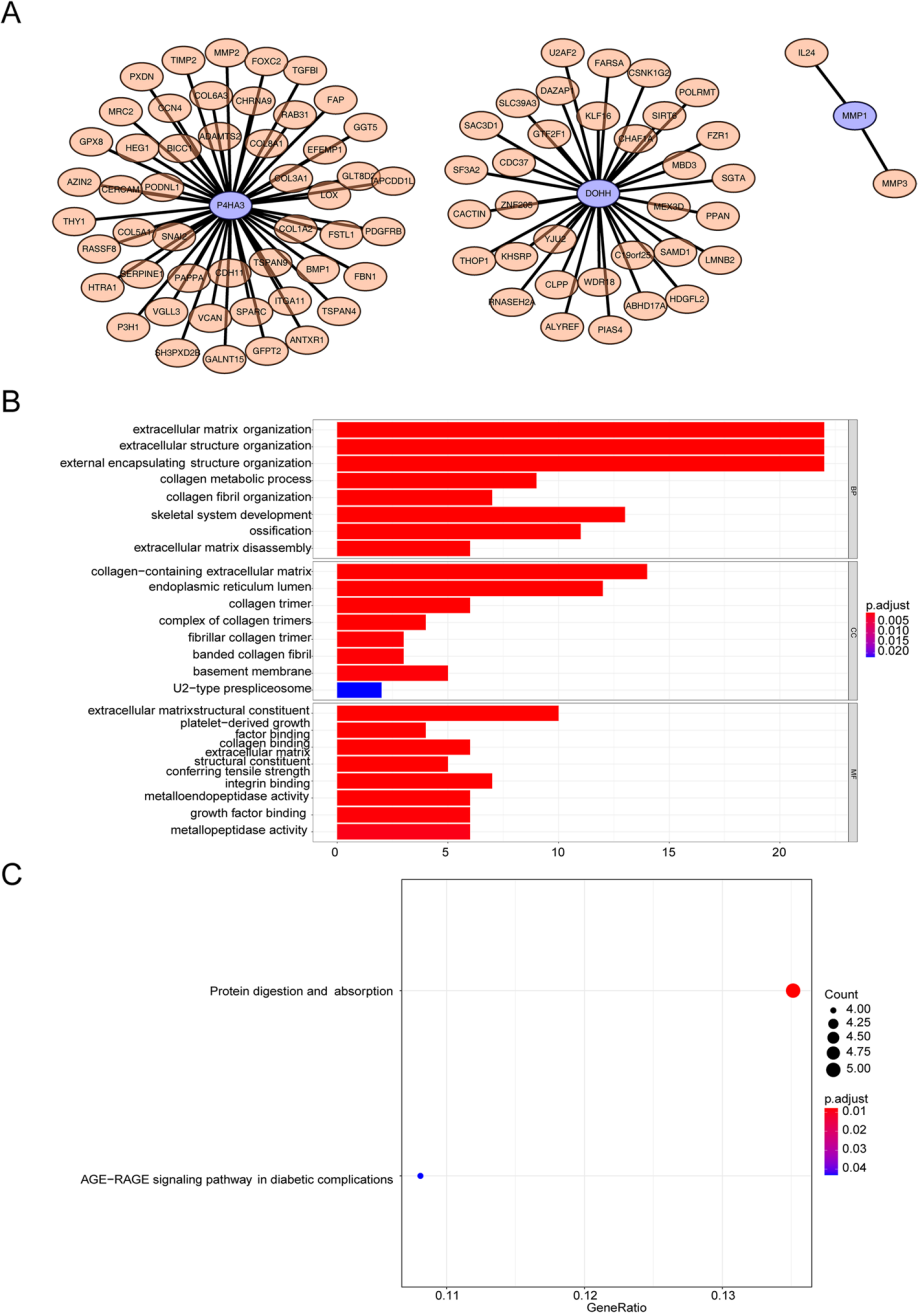
The network, KEGG and GO analyses of collaborative genes. **A** The PPI (Protein–Protein Interaction) network of signature genes; **B** GO analysis on BP, CC, and MF; **C** KEGG analysis on the enrichment pathway of collaborative genes

To investigate genes that act synergistically with signature genes, the pearson correlations were calculated between pairwise genes with the criteria of |Cor|> 0.6, FDR < 0.05 (Fig. 9A). The *P4HA3* were associated with 49 genes, such as *ADAMTS2*, *ANTXR1* and *APCDD1L* (Fig. 9A). The *DOHH* were associated with 33 genes, such as *ADAMTS2*, *ANTXR1* and *APCDD1L* (Fig. 9A). The *MMP1* were associated with 2 genes including *IL24* and *MMP3* (Fig. 9A). The GO and KEGG results of 84 collaborative genes were shown in the Fig. 9B-C. The top 8 GO terms were collagen-containing extracellular matrix and extracellular matrix organization etc. The KEGG pathways of enrichment were ‘Protein digestion and absorption’ as well as ‘AGE-RAGE signaling pathway in diabetic complications’.

### Construction of the TF-miRNA-Gene network targeting signature genes

**Fig. 10:**
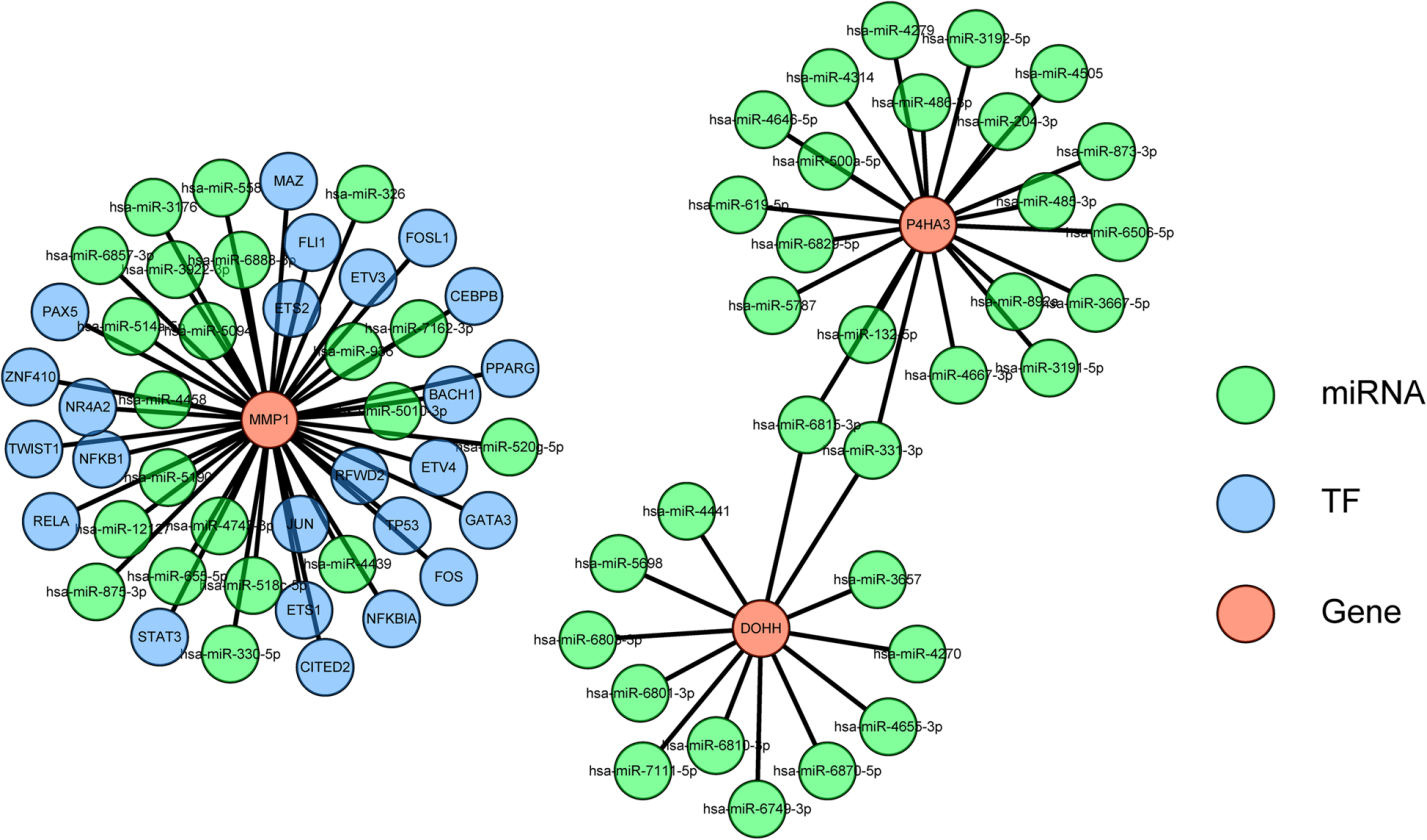
The Transcription factors (TFs)-miRNA-mRNA network targeting signature genes. Red represents the signature gene, green represents the miRNAs, blue represents the TFs

In order to further elaborate the upstream regulatory miRNAs and TFs effecting signature genes, the TF-miRNA-mRNA network was predicted and pictured in Fig. 10, where 24 TF targeting *MMP1* (such as NF-κB, STAT3, ETS1, CITED2, BACH1) as well as 53 miRNAs interacting with three signature genes were predicted. It can be seen that the hsa-miR-6815-3p and hsa-miR-331-3p were common to regulate the expression of *P4HA3* and *DOHH*.

### qRT-PCR

**Fig. 11:**
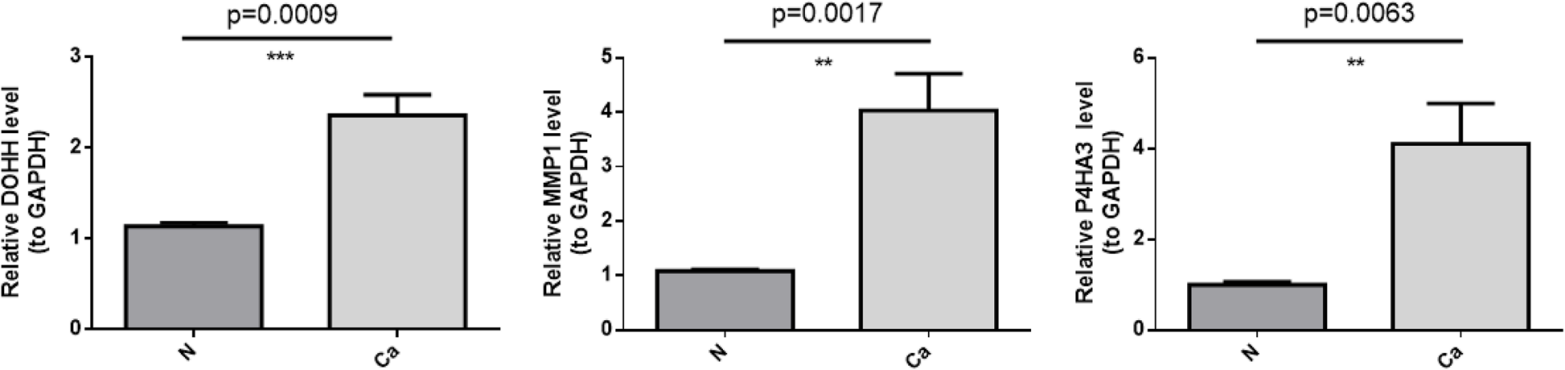
Results of qRT-PCR for the expression of *DOHH*, *P4HA3* and *MMP1*

The expression of *DOHH*, *P4HA3* and *MMP1* were detected via qRT-PCR. The abundance of these genes were higher in tumor cases than normal cases (Fig. 11).

## Discussion

In order to investigate the prognostic IMRGs signature in GC, *DOHH*, *P4HA3* and *MMP1* was selected using univariate and multivariate Cox regression analyses. The Kaplan Meier curves manifested the worse outcome of patients in the high-risk group. ROC curves confirmed that the IMRGs signature presented good efficiency for predicting GC prognosis. Further, the positive correlation of *P4HA3* and *MMP1* expression as well as the negative correlation of *DOHH* expression with risk score and worse prognosis were detected as well, and the *MMP1* expression significantly increased in the cohorts at age > 60 and Stage II-IV. Finally, qRT-PCR revealed that the abundance of *DOHH*, *P4HA3* and *MMP1* were high in tumor cases, indicating the complex mechanism between the high expression of *DOHH* as a protective factor and the high expression of *P4HA3* and *MMP1* as the risk factors in the development of GC. We make the case that this study has valuable significance for foretelling the OS of GC.

Iron is required for cell proliferation and growth, and it promote the formation of toxic-free radical species. Besides, iron metabolism-relevant pathways, containing uptake-export, storage, and regulation processes, may be aberrantly mediated during the course of cancer progression [21]. For instance, transferrin receptor 1 (TFR1), which is a carrier in the regulation of cell growth as well as iron uptake, is aberrantly expressed in tumors and is intimately linked to tumour proliferation as well as metastasis [22–24]. This has led to TFR1 being an ideal target for cancer prevention and cure [25]. Besides, a recent study on GC shows that the combination of amino acid, lipid, and iron metabolism might play a role in malignancies by participating in ferroptosis-related metabolic regulation mechanisms, and the prediction model targeting ferroptosis in GC has also been widely studied [26]. Prior investigations have shown that iron metabolism probably contribute to a number of cancers, namely lung cancer [27], leukemia [28], prostate cancer [29], and kidney cancer [30]. Nevertheless, present researches has centered on the involvement of iron metabolism in cancer progression and management, with hardly ever discussing the value of iron metabolism-related genes in cancer prognosis foretelling. Thus, the prognostic signature of iron metabolism in GC required to be adequately probed.

As a HEAT-repeat protein, DOHH has eight tandem helical hairpins on a symmetric dyad. DOHH encompasses two possible iron coordination sites (one on each dyad) which comprised of two rigorous conserved His-Glu motifs, and the activity of DOHH activity could be recovered nothing but by the appending of Fe^2+^ to the apoenzyme [31]. DOHH catalyzes the last process of maturation of eIF5A, an momentous protein in proliferation of eukaryotic cell [32–35]. The metal chelating compounds could efficiently suppress deoxyhypusine hydroxylation in eIF5A, and arrest the progression of cell cycle in mammalian cells, including human cancer cells and HUVEC cells, at the boundary of G1/S [35, 36]. *P4HA3* is expressed at very low levels in normal adults and fetal tissues [37]. A recent research relying on the bioinformatic analysis and TCGA database found that up-regulation of *P4HA3* was highly linked to genes responding to ECM generation in breast cancer, and higher expression of *P4HA3* is relevant to worse prognosis [38]. Previous studies have shown *P4HA3* is significantly up-regulated in GC, and up-regulation of *P4HA3* is epigenetically activated by Slug, which is correlated with GC metastasis and poor survival [39]. MMP-1 have been authenticated to have agonist activity against PAR1, which is expressed in most human tissues, containing the majority of cell types in the blood vessel wall, platelets, and inflammatory cells, and is thought to be the primary enzyme responsible for collagen degradation [40–44]. Although basal expression of *MMP-1* is widespread, some disease states lead to further increasing expression of *MMP-1*, a result that is usually linked to adverse outcomes. Combined with these studies, the prognosis prediction of GC was composed of three iron metabolism related genes, including *DOHH*, *P4HA3* and *MMP1*. The external verification set further proved the good performance of three gene signature in predicting GC prognosis Jianming Wei et al. found that *LC22A17* associated with poor overall survival in GC [45]. Yuehong Cui et al. found that NOTCH1 and NOTCH3 associated with poor overall survival and low expression of NOTCH2 associated with poor overall survival in GC [46]. In the current research, we postulated that the iron metabolism-relevant signature for OS can adequately foretell the clinical consequences of GC patients.

The concept of immunity promoting or suppressing the tumours is widely accepted, and one of the most influential anticancerous therapies that have been exploited in these years is the therapy targeting immune checkpoints. And the development of tumours is not only regulated by the intrinsic variations in cancerous cells but also dependent on the lymphocyte infiltration and activation [47]. Our results uncovered that the immune state was notably disparate between the two risk GC patients, containing the the proportions of Macrophages, dendritic cells (DCs), Eosinophils, Immature dendritic cells (DCi), Neutrophils, Natural killer cells (NK), Mast cells, Plasmacytoid dendritic cells (DCp), Tem, Gamma delta T cells (γδT) and Thi cells. Huang XM et al. found that ICOS( +) Tregs associated with poor overall survival in GC and pDCs play a underlying role in recruiting ICOS( +) Tregs [48]. Sammarco G et al. found that mast cells exerted a cancer promotion role in GC through the release of angiogenic and lymphangiogenic factors [49]. Eosinophils have either anti-tumor impacts or stimulate the development of tumour by secreting various cytokines and factors containing eosinophil-derived neurotoxin, peroxidase, eosinophil cationic protein, and major basic protein [50]. Macrophages was comprised of two main types, namely M1 and M2. Macrophage M2 exert an momentous role in tumor progression, facilitating pro-angiogenic and immunosuppressive signal in the tumor while M1 macrophage infiltration may be linked to a favorable survival rate [51]. People suffering from GC displayed a notably higher neutrophil infiltration in GC tumuor tissues. These tumour-infiltrating neutrophils revealed a phenotype of activated CD54^+^ and expressed high level programmed death-ligand 1 (PD-L1), which was an immunosuppressive molecule and relevant to disease progression and lowered GC patient survival [52]. As vital lymphocytes in innate immunity, NK cells exert important impact in restraining GC initiation, progression, and metastases, and can improve NK cells’ killing activity toward GC. Gene therapy have been uncovered to directly or indirectly activate NK cells [53]. The memory T cell (Tm) comprises of two populations, namely the effector memory T cells (Tem) and central memory T cells (Tcm), and the ratio of CD4( +)/CD8( +) Tem were notably increased in GC than healthy controls [54]. In addition, the prognostic genes (*DOHH*, *P4HA3* and *MMP1*) have remarkable linkages with immune cell, suggesting the complexity between iron metabolism and immunity. Moreover, GO and KEGG analyses performed using these 84 collaborative genes suggested that these 84 collaborative genes were primarily engaged in ‘AGE-RAGE signaling pathway in diabetic complications’ and ‘protein digestion and absorption’. In fact, previous studies revealed that ‘AGE-RAGE signaling pathway in diabetic complication constitute the main mechanisms of vascular oxidative stress and enable the activation of NADPH oxidase (Nox) and NF-κB, thus incepting a vicious cycle of oxidative stress and inflammation [55–60]. Previous studies have demonstrated that the receptor activator of NF-κB ligand signaling pathway can promote the metastasis of tumor cells [61, 62]. The ‘protein digestion and absorption’ pathway had been found to be linked to pancreatic neuroendocrine tumours as well as breast cancer [63, 64]. Expression of *DOHH*, *P4HA3* and *MMP1* was assessed with qRT-PCR in tumor cases and the normal cases. The results demonstrated that the abundance of *DOHH*, *P4HA3* and *MMP1* were higher in tumor cases than normal cases. The experiment verified the aforementioned conclusion.

All in all, this study manifested that the IMRGs (*DOHH*, *P4HA3* and *MMP1*) presented good efficiency for predicting GC prognosis, implying that this gene signature relevant to iron metabolism was a promising biomarker in foretelling the prognosis of GC, which would provide new idea on the digging of underlying predictive biomarkers for GC patients. Nevertheless, this study had certain limitations. For instance, public data may have certain boundedness when we analyze the prognostic performance of gene signatures. Additional data, including primary data from patients with GC, are required to affirm the predictive effect of these gene signature.

### Supplementary Information

**Additional file 1:** **Table S1.** Primer information.

## Data Availability

The datasets analysed during the current study are available in the TCGA repository, [https://portal.gdc.cancer.gov/projects/TCGA-STAD]. The mRNA-seq datasets generated during the current study are available from the corresponding author on reasonable request.

## Abbreviations

GC: Gastric cancer
IMRGs: Iron metabolism-relevant genes
DE-IMRGs: Differentially expressed IMRGs
ROC: Receiver operating characteristic
ssGSEA: Single-sample GSEA
AUCs: Area under the ROC curves
OS: Overall survival
MSigDB: Molecular Signatures Database
DEGs: Differentially expressed genes
TFR1: Transferrin receptor 1
TCGA: The Cancer Genome Atlas
DC: Dendritic cells
DCi: Immature dendritic cells
NK: Natural killer cells
DCp: Plasmacytoid dendritic cells
ΓδT: Gamma delta T cells
PD-L1: Programmed death-ligand 1
Tm: Memory T cell
Tem: Effector memory T cells
Tcm: Central memory T cells
Nox: NADPH oxidase
TFs: Transcription factors
qRT-PCR: Quantitative real-time PCR
GSEA: Gene Set Enrichment Analysis
