# Supplementary material for: Construction of iron metabolism-related prognostic features of gastric cancer based on RNA sequencing and TCGA database
Source: BMC Cancer. 2023 Nov 13;23:1106. doi: 10.1186/s12885-023-11569-9 (PMC10644585; doi:10.1186/s12885-023-11569-9)
Supplement: Supplementary file 1 — Additional file 1: Table S1. Primer information. [file 12885_2023_11569_MOESM1_ESM.docx]

**Table S1.** Primer information

| **Gene** | **Reverse Primer sequence** |
| --- | --- |
| DOHH F | ACGGAGCAGGAGGTGGAT |
| DOHH R | GCTTGAGCAGGGCGGAAT |
| P4HA3 F | CCCTCTGCTTGCATTTACTC |
| P4HA3 R | GTCTTGCTCCACCTTCTCAT |
| MMP1 F | CTCTGGAGTAATGTCACACCTCT |
| MMP1 R | TGTTGGTCCACCTTTCATCTTC |
| GAPDH F | CCCATCACCATCTTCCAGG |
| GAPDH R | CATCACGCCACAGTTTCCC |
